# Dr. Theodore P. Knapp

**Published:** 1913-03

**Authors:** 


Dr. Theodore P. Knapp, Hahnemann of Philadelphia, 1854, died at his home in Union, Broome County, N. Y., September 25, 1912. He had been in practice in Union since 1859.

